# Supplementary material for: Widespread expression of the ancient HERV-K (HML-2) provirus group in normal human tissues
Source: PLoS Biol. 2022 Oct 18;20(10):e3001826. doi: 10.1371/journal.pbio.3001826 (PMC9578601; doi:10.1371/journal.pbio.3001826)
Supplement: S1 Fig — This figure displays a heatmap of individual HML-2 expression from 2 female GTEx donors. Provirus expression is given in TPM for each provirus detected (10 in Donor 1 and 8 in Donor 2) for 22 body sites. Each provirus is labeled on the side with which donor it was measured in. This heatmap was made using data from S1 Data. GTEx, Genotype Tissue and Expression; HML, human mouse mammary tumor virus-like; TPM, transcripts per million. (PDF) [file pbio.3001826.s001.pdf]

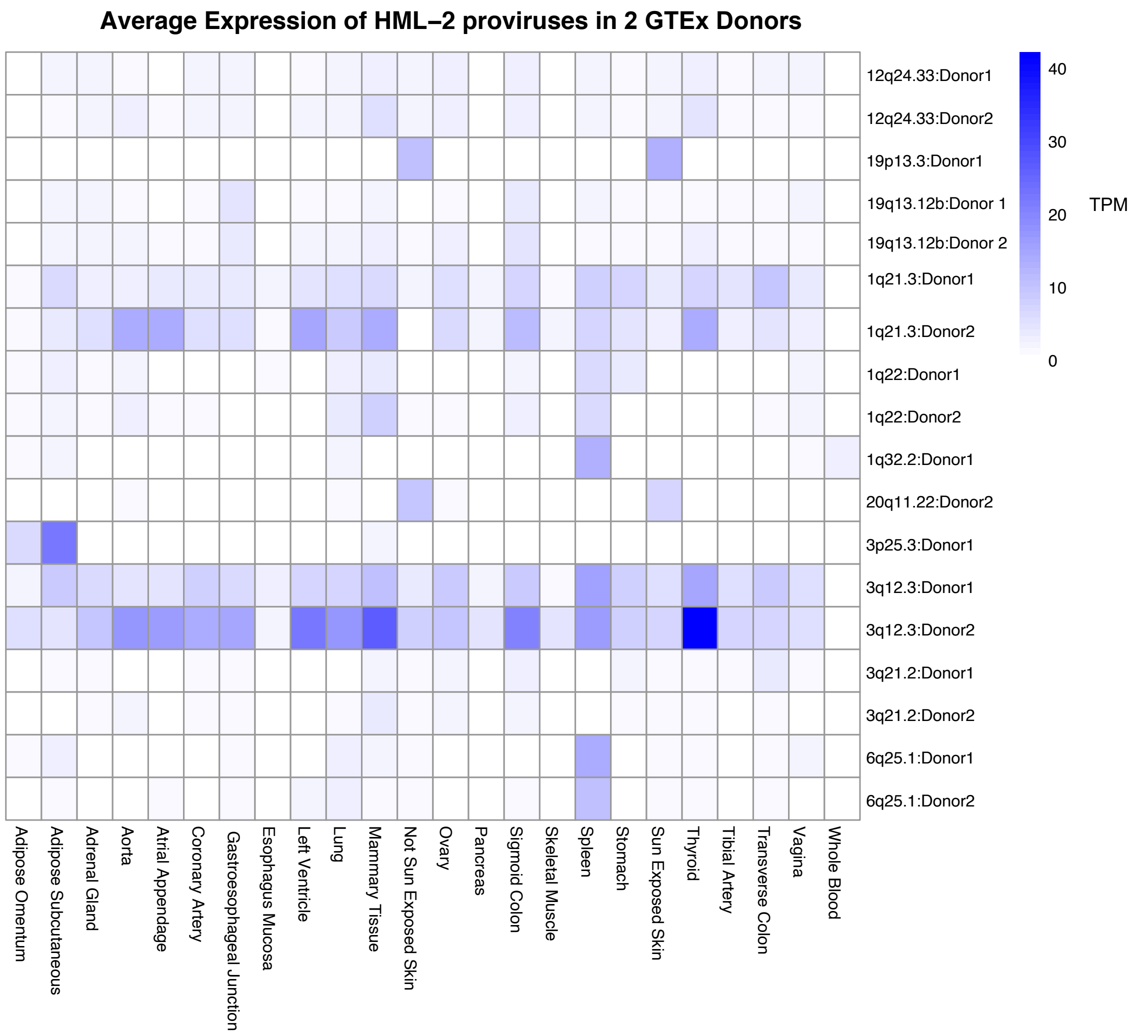

Supplemental Figure 1. Comparison of individual donors
This figure displays a heatmap of individual HML-2 expression from two female GTEx donors. Provirus expression is given in TPM for each provirus detected (10 in Donor 1 and 8 in Donor 2) for 22 body sites. Each provirus is labeled on the side with which donor it was measured in. This heatmap was made using data from S2_Data.
