# Supplementary material for: Widespread expression of the ancient HERV-K (HML-2) provirus group in normal human tissues
Source: PLoS Biol. 2022 Oct 18;20(10):e3001826. doi: 10.1371/journal.pbio.3001826 (PMC9578601; doi:10.1371/journal.pbio.3001826)
Supplement: S2 Fig — This figure displays the alignment of RNA-seq reads to the provirus 22q11.23 in a prostate sample visualized in Integrated Genomics Viewer. The sequence of 22q11.23 is defined by the vertical black lines. The LTR5HS LTR is displayed upstream to the left of the black line. The blue bars shown above the image indicate repeat elements as defined by the Repeatmasker track for HG38 downloaded from UCSC. The bam file and index file for this screenshot can be found in S4 Data. (PDF) [file pbio.3001826.s002.pdf]

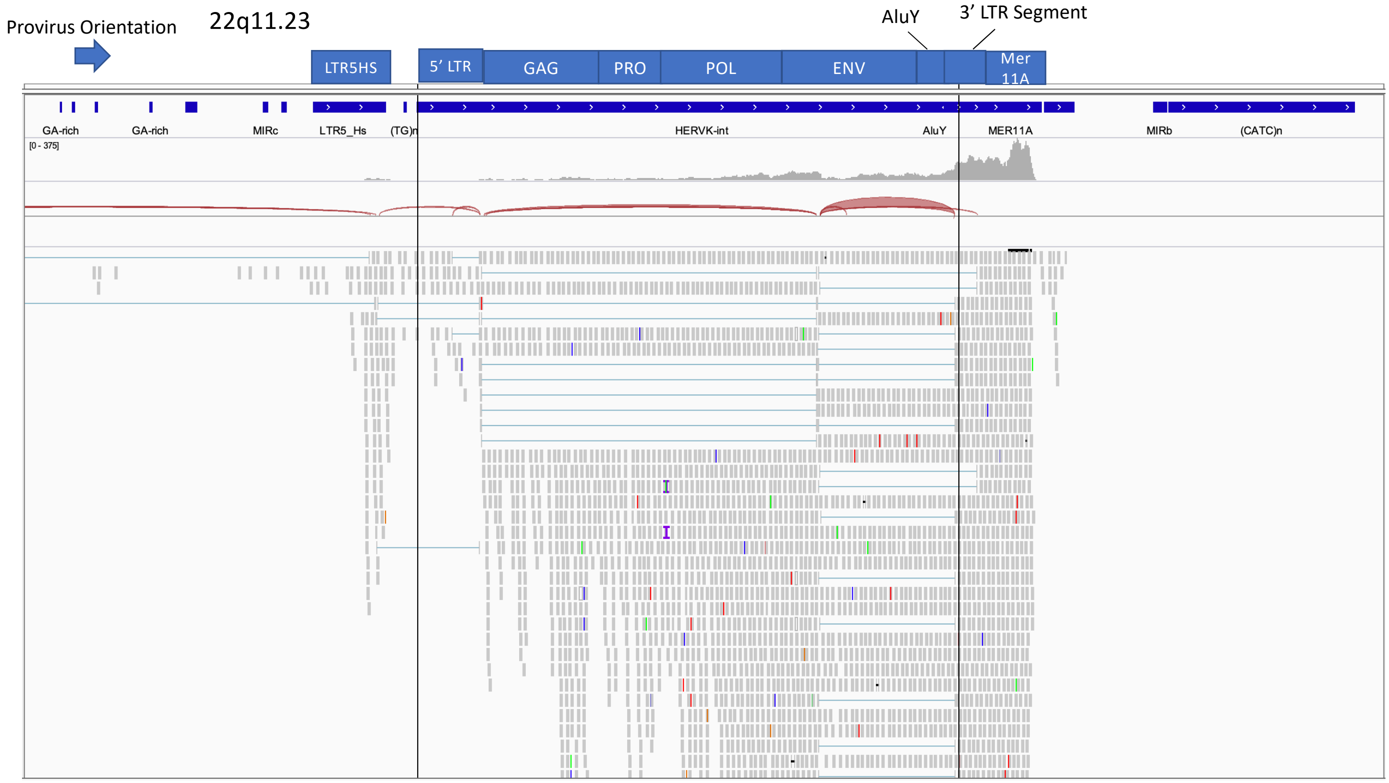

Supplemental Figure 2. 22q11.23 IGV Screenshot

This figure displays the alignment of RNA-seq reads to the provirus 22q11.23 in a prostate sample visualized in Integrated Genomics Viewer. The sequence of 22q11.23 is defined by the vertical black lines. The LTR5HS LTR is displayed upstream to the left of the black line. The blue bars shown above the image indicate repeat elements as defined by the Repeat-masker track for HG38 downloaded from UCSC. The bam file and index file for this screenshot can be found in S5_Data.
