# Supplementary material for: Widespread expression of the ancient HERV-K (HML-2) provirus group in normal human tissues
Source: PLoS Biol. 2022 Oct 18;20(10):e3001826. doi: 10.1371/journal.pbio.3001826 (PMC9578601; doi:10.1371/journal.pbio.3001826)
Supplement: S3 Fig — This figure displays a scatterplot of expression of both 11q12.3 and ASRGL1 expression in 166 prostate samples in the GTEx dataset. Outliers more than 1.5 times beyond the upper and lower quartiles were removed. Smoothing line was added using linear model method in R ggplot2. A 0.95 confidence interval is displayed around the line. This figure was generated using data from S1 Data. (PDF) [file pbio.3001826.s003.pdf]

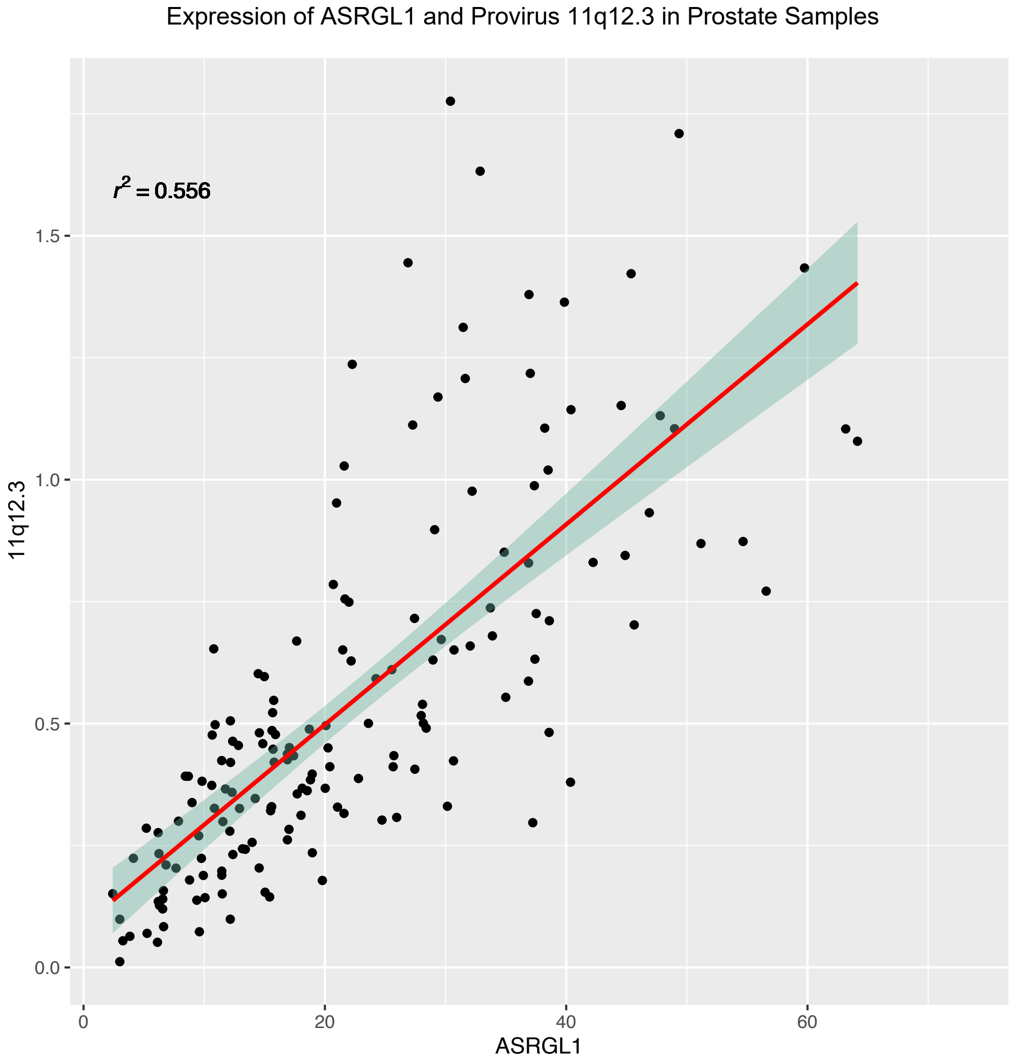

`

Supplemental Figure 3 Scatterplot of ASRGL1 and 11q12.3 in Prostate tissue
This figure displays a scatterplot of expression of both 11q12.3 and ASRGL1 expression in 166 Prostate samples in the GTEx dataset. Outliers more than 1.5 times beyond the upper and lower quartiles were removed. Smoothing line was added using linear model method in R ggplot2. A 0.95 confidence interval is displayed around the line. This figure was generated using data from S2_Data.
